# Evaluation of video compression methods for cone‐beam computerized tomography

**DOI:** 10.1002/acm2.12596

**Published:** 2019-05-09

**Authors:** Hui Yan, Yexiong Li, Jianrong Dai

**Affiliations:** ^1^ Department of Radiation Oncology Cancer Hospital Chinese Academy of Medical Sciences Beijing China

**Keywords:** cone‐beam computerized tomography, image compression, JPEG, MPEG, video compression

## Abstract

**Purpose:**

Cone‐beam computerized tomography (CBCT) is routinely performed for verification of patient position in radiotherapy. It produced a large amount of data which require a method to compress them for efficient storage. In this study three video compression algorithms were introduced and their performance was evaluated based on real patient data.

**Materials and methods:**

At first CBCT images in multiple sets of a patient were transferred from reconstruction workstation or exported from treatment planning system. Then CBCT images were sorted according to imaging time (time‐prioritized sequence) or imaging location (location‐prioritized sequence). Next, this sequence was processed by a video compression algorithm and resulted in a movie. Three representative video compression algorithms (Motion JPEG 2000, Motion JPEG AVI, and MPEG‐4) were employed and their compression performance was evaluated based on the CBCT data of 30 patients.

**Results:**

Among three video compression algorithms, Motion JPEG 2000 has the least compression ratio since it is a lossless compression algorithm. Motion JPEG AVI and MPEG‐4 have higher compression ratios than Motion JPEG 2000 but come with certain image losses. For MPEG‐4, location‐prioritized sequences show higher compression ratio than time‐prioritized sequences. Based on the results achieved on the clinical target verification application, the registration accuracy of CBCT after decompression was comparable to that of the original CBCT.

**Conclusions:**

Video compression algorithms could provide a higher compression ratio comparing to static image compression algorithm. Although the loss of CBCT image due to compression its impact on registration accuracy of patient positioning is almost negligible. Video compression method is an effective way to substantially reduce the size of CBCT images for storage.

## 1 INTRODUCTION

Medical imaging plays a key role in modern medicine since they offer comprehensive information for diagnosis, treatment, and follow‐up. However, the amount of data generated by the imaging procedure is exploding and causes higher cost to store them. Besides the demand of storage, there are many situations that the amount of data must be reduced such as low‐speed network connection, low‐resolution presentations, and printings.^1^, ^2^ Image compression will reduce the file size on the storage device while maintaining relevant clinical information. Image compression algorithm takes advantages of redundancy that occur spatially, temporally, and spectrally. It can be categorized in lossy and lossless techniques.^3^ Lossless techniques are reversible and compression rates are low. Lossy techniques are irreversible and compression rates are much higher. Because of the regulatory policies set by agencies, there is few clinical research on the use of lossy compression for medical images.^4^ In contrast, the lossless compression for medical images adopted by many organizations and standards in medicine, such as the Digital Imaging and Communications in Medicine (DICOM) group.^5^, ^6^, ^7^, ^8^

Cone‐beam computed tomography (CBCT) is a routine imaging procedure for verification of patient position in radiotherapy.^9^ As designed for daily use, many sets of CBCT images are resulted during patient setup of multi‐faction IMRT.^10^, ^11^, ^12^ Due to low soft tissue contrast, CBCT is usually used for patient setup and rarely for treatment planning purpose. In clinical practice, daily CBCT images are reconstructed on local workstation and saved in the form of DICOM images in a folder. CBCT data will grow quickly in reconstruction workstation due to the large amount of patients under image‐guided radiotherapy (IGRT) and cause dysfunction of the other services or processes in the local computer. Therefore, CBCT data need to be backed up to a storage equipment in a relatively higher frequency to free more space in the local computer.

For efficient storage of CBCT data for clinical service and research purpose, the high‐performance image compression method is needed in order to reduce CBCT data on disk. Since CBCT image sets are acquired in the same setup position of patient, the correlation between them are higher and could be utilized to reduce data redundancy for compression purpose.^13^, ^14^ In this study three video compression algorithms were employed for CBCT data. Analogous to motion pictures, CBCT images are rearranged in a sequence and processed by three video compression algorithms. In Section 2, the standards of image and video compression are briefly introduced and followed by the principle of video compression. Next, the workflow of video compression algorithm for CBCT images was explained. In Section 3, the performance of three video compression algorithms was compared based on clinical data. Finally, the merits and disadvantages of the video compression method were discussed.

## 2 MATERIALS AND METHODS

### 2.1 Standards of image and video compression

There are many working groups that aim at image and video coding. They formed many working groups dedicated to set standard for image, audio, and video compression and transmission.^15^, ^16^, ^17^ The most famous standards are JPEG, MPEG, and H.26x.

The Joint Photographic Experts Group (JPEG) was created in 1986 and is the joint committee between International Organization for Standardization (ISO)/International Electronic Commission (IEC) and International Telegraph and Telephone Consultative Committee (CCITT) that created the JPEG and JPEG 2000 standards.^18^ JPEG commonly used lossy compression algorithm for digital images, particularly for those images produced by digital photography. JPEG 2000 was created in 2000 with the intention of superseding the original discrete cosine transform (DCT)‐based JPEG standard with wavelet‐based method. Motion JPEG 2000 (MJ2) is a file format for motion sequences of JPEG 2000 images. It is intrinsically intra‐frame coding algorithm which means each frame is coded independently.

To meet the demand of video compression, the Moving Picture Experts Group (MPEG) was created by CCITT and ISO in 1988 to set standards for motion image compression and transmission. MPEG implements inter‐frame coding that means each frame is coded in predictive mode. MPEG‐1 is the first MPEG compression standard for audio and video. MPEG‐2 is video and audio standard for broadcast‐quality television. MPEG‐3 (MP3) was intended for HDTV compression and was merged with MPEG‐2. MPEG‐4 (MP4) uses further coding tools with additional complexity to achieve higher compression factors than MPEG‐2, and is still an evolving standard.

The Visual Coding Experts Group was created in 1984 and is the study group of the International Telecommunication Union (ITU) Telecommunication Standardization Sector. It is responsible for standardization of the “H.26x” line of video coding standards, the “T.8xx” line of image coding standards, and related technologies. H.261 was the first practical digital video coding standard. H.263 was developed as an evolutionary improvement based on experience from H.261, and the MPEG‐1 and MPEG‐2 standards.^19^ H.264 is the most widely used standard in the series of international video coding standards.^20^

### 2.2 Principle of image and video compression

The goal of image compression is to reduce image size for storage and transmission without losing relevant image information. There are certain redundancies existing in images including space, time, structure, etc. Image compression algorithms utilize these redundancies for the best compression ratio. For static image, there are hundreds of compression algorithms and categorized into lossless encoding and lossy encoding methods. The most popular compression algorithms in the medical image community are lossless JPEG and lossless JPEG 2000. JPEG and JPEG 2000 have been adopted by DICOM in 2001. Motion JPEG and Motion JPEG 2000 provide a file format for sequence of JPEG and JPEG 2000 images. They are intended to coexist with MPEG. For motion image, the compression algorithms can be categorized into inter‐frame prediction coding, three‐dimensional transformation coding, mode‐based coding, etc. Inter‐frame prediction coding utilizes the strong correlation between successive frames and is the most popular video compression algorithm which was adopted by many international standards such as MPEG‐4 and H.264.

**Figure 1:**
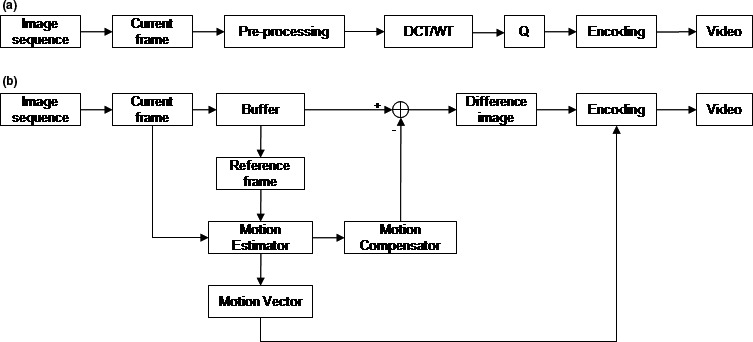
The block diagram of (a) intra‐frame compression algorithm and (b) inter‐frame compression algorithm.

Video encoding technique exploits certain characteristics of video signals, namely, redundancy of information both intra‐frame (spatial redundancy) and inter‐frame (temporal redundancy).^21^ The intra‐frame compression algorithm (Figure 1a), such as Motion JPEG or Motion JPEG 2000, begins by calculating the DCT or wavelet transform (WT) coefficients over small image blocks. This block‐by‐block processing takes advantage of the image's local spatial correlation properties. The DCT or WT process produces many 2D blocks of transform coefficients that are quantized (Q) to discard some of the trivial coefficients. The quantized coefficients are then processed by encoding to form a video frame. On the other hand, inter‐frame coding [Fig. 1(b)], such as MPEG, exploits temporal redundancy by predicting future frames from previous reference frames. The motion estimator searches reference frames for areas similar to those in the current frame. This search results in motion vectors, which is used to form a prediction of the current frame based on reference frames via motion compensator. The difference image between the current frame and predicted frame is then calculated. Since only difference image and motion vector needed to be encoded instead of the original image, inter‐frame coding always results in a significant reduction in video size.

### 2.3 Video compression for CBCT images

**Figure 2:**
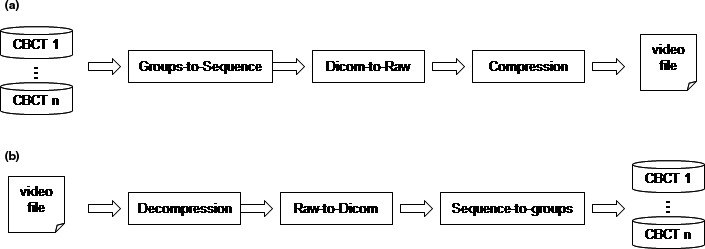
The workflows of (a) video compression process and (b) video decompression process.

The work flow of video compression process for CBCT images is shown in Fig 2(a). It consists of three steps: (a) CBCT image in multiple sets are sorted according to their imaging time or location, and put together in a time‐prioritized or location‐prioritized sequence; (b) image sequence in DICOM format are then converted to image sequence in raw‐data format; (c) the image sequence in raw‐data format is later processed by compression program and a movie file is achieved. Decompression is the reverse process of compression process. The work flow of video decompression process for CBCT images is shown in Fig 2(b). It consists of three steps: (a) images in raw‐data format are extracted from video file by decompression algorithm; (b) the image sequence in raw‐data format are then converted to image sequence in DICOM format; (c) CBCT images are later identified according to imaging time and location, and grouped into multiple sets they are originally from.

**Figure 3:**
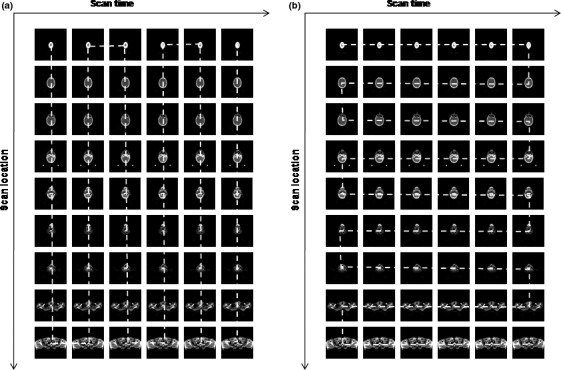
The illustration of cone‐beam computerized tomography images in (a) time‐prioritized sequence and (b) location‐prioritized sequence.

It is important to find an effective way to combine all CBCT images of multiple sets into a single sequence for the best compression performance. The step (a) of compression process was implemented in two ways based on imaging time and location of CBCT images. The sequence sorted based on imaging time or generation time are called time‐prioritized sequence, while the sequence sorted based on imaging location or slice location are called location‐prioritized sequence. The difference between two image sequences is illustrated in Fig. 3. The columns represent CBCT sets obtained in six imaging sessions while the rows represent nine successive slice locations in a patient body. Dotted line indicates the direction in which the previous image connects to the next image in a sequence. Images are connected along the column direction in time‐prioritized sequence because columns are sorted by generation times as shown in Fig. 3(a), while images are connected along the row direction in location‐prioritized sequence because rows are sorted by slice locations as shown in Fig. 3(b). There are more ways to sorting CBCT images in a sequence and the two mentioned above are straightforward and easy to implement.

### 2.4 Evaluation

The performance of three video compression algorithms as mentioned above was investigated in this study. They are Motion JPEG 2000 (MJ2), Motion JPEG AVI (AVI), and MPEG‐4 (MP4). The function of three algorithms is provided by VideoWriter of Matlab (MathWorks, Inc. Natick, MA, USA). For MJ2, compression ratio is not specified in advance to allow video data to be compressed as much as possible. For AVI and MP4, the video quality is set to its maximum number 100 to allow the best quality of video. These tests were performed on a personal computer equipped with Intel i7 CPU 2.4‐GHz and 12 GB RAM. The programs for data processing were developed with Matlab (version 2013). The CBCT images were collected from 30 patients with treatment sites at head and neck (10 cases), thorax (10 cases), and pelvis (10 cases). For each patient, 8‐10 CBCT sets are used for testing.

The performance of video compression algorithm was evaluated by compression ratio and compression time. Compression ratio is defined as the ratio between the file sizes of image sequence and video file. Compression time is the average time for processing one image and calculated by the total compression time dividing the total number of images processed.

The similarity in quality of image sequence is evaluated by difference and correlation between all successive images in a sequence. Higher value of similarity of a sequence means there is more redundant information to be reduced and larger compression ratio is expected. The image difference (DIFF) is calculated by mean value of image differences in a sequence as defined below.

(1) $$\text{DIFF}=\frac{1}{L-1}\left\{\sum_{k=1}^{L-1}\left(\frac{\sum_{i=1}^{M}\sum_{j=1}^{N}\left|{\text{CBCT}}_{\mathrm{ijk}}^{}-{\text{CBCT}}_{ijk+1}^{}\right|}{M\times N}\right)\right\}$$

The image correlation (CORR) is calculated by mean value of image correlation coefficients in a sequence as defined below.

(2) $$\begin{array}{cc} & \text{CORR}=\frac{1}{L-1}\sum_{k=1}^{L-1}R\left({\text{CBCT}}_{k}^{},{\text{CBCT}}_{k+1}^{}\right)\\ & \mathrm{where}\overset{}{}R\left(A,B\right)=\frac{\sum_{i=1}^{M}\sum_{j=1}^{N}\left(A_{\mathrm{ij}}^{}-\bar{A}\right)\left(B_{\mathrm{ij}}^{}-\bar{B}\right)}{\sqrt{\sum_{i=1}^{M}\sum_{j=1}^{N}{\left(A_{\mathrm{ij}}^{}-\bar{A}\right)}^{2}}\sqrt{\sum_{i=1}^{M}\sum_{j=1}^{N}{\left(B_{\mathrm{ij}}^{}-\bar{B}\right)}^{2}}} \end{array}$$

The performance of video decompression algorithm is evaluated by decompression time, mean square error (MSE), peak signal‐to‐noise ratio (PSNR), and video quality matrix (VQM). Decompression time is the average time for processing one image from video. The MSE is calculated by comparing original and decompressed images pixel by pixel as defined below.

(3) $$\text{MSE}=\frac{1}{L}\left\{\sum_{k=1}^{L}\left(\frac{\sum_{i=1}^{M}\sum_{j=1}^{N}{\left({\text{CBCT}}_{\mathrm{ijk}}^{\mathrm{decompressd}}-{\text{CBCT}}_{\mathrm{ijk}}^{}\right)}^{2}}{M\times N}\right)\right\}$$

It is important to compare the error of an image with respect to the amount of bits a pixel is encoded. PSNR is the ratio between the maximum power of a signal and the power of corrupting noise that affects the fidelity of its representation. In this case it is defined as:

(4) $$\text{PSNR}=10.\log_{10}\left(\frac{{\text{MAX}}^{2}}{\text{MSE}}\right)\left[dB\right]$$

here, *MAX* is the maximum possible pixel value of the image and 2^16^‐1 in this study. Typical values for the PSNR of lossy image and video compression are between 60 and 80 dB, provided the bit depth is 16 bits. VQM is a metric to predict human‐perceived video quality and is defined as:

(5) $$\text{VQM}=\frac{1}{1+e^{\alpha (\text{PSNR}-b)}}$$

The values of a = 0.15 and b = 19.7818 have been set experimentally. The resulting VQM is compared to fuzzy results like “excellent” (VQM < 20%) or “good” (VQM < 40%).^22^

**Figure 4:**
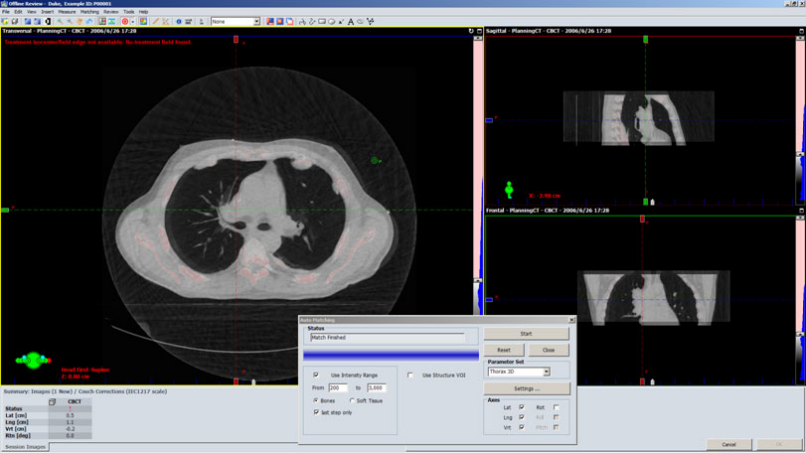
The illustration of cone‐beam computerized tomography registration for lung tumor patient based on bony structures.

**Figure 5:**
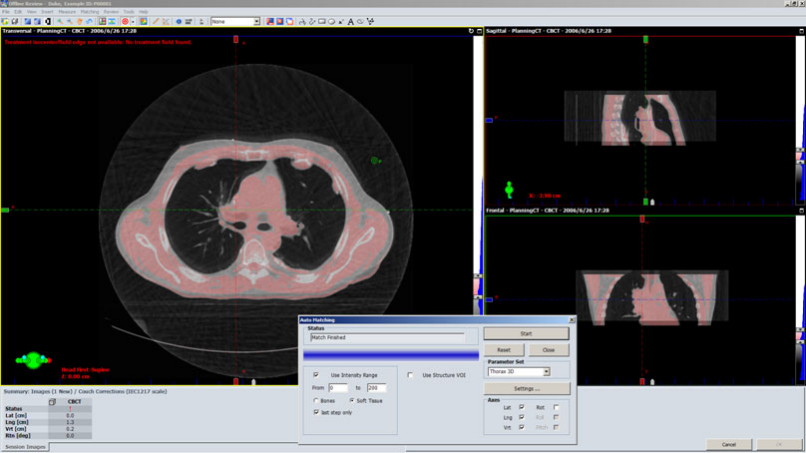
The illustration of cone‐beam computerized tomography registration for lung tumor patient based on soft tissues.

The impact of image loss on positioning accuracy was assessed using a clinical image registration application — offline review (Varian medical system, Palo Alto, CA, USA). First, the original CBCT images were automatically registered with planning CT to determine target offset for patient positioning. Next, CBCT images were compressed to a video and then decompressed from video to another set of CBCT images. The CBCT images after decompression were automatically matched with planning CT to determine another target offset. The difference between both sets of target offsets is the discrepancy caused by image loss due to compression algorithm. This discrepancy represents the inconsistency of registration accuracy before and after compression. The image registration was performed automatically and the parameters were set for bony structures and soft tissues, respectively, as shown in dialog window of Figs. 4 and 5. Specifically, the intensity ranges for bony structure and soft tissues were set to 0–200 and 200–3000. For each session, the target offsets in three dimensions were displayed at the left‐bottom corner of main application.

## 3 RESULTS

### 3.1 Video compression

The performance of three video compression algorithms was compared as shown in Table 1. Compression ratio and running time are reported for three algorithms. The similarity quality metrics (DIFF and CORR) are reported for two sequences of MP4. For each treatment site, the CBCT data of 10 patients were analyzed for each site and the mean values are shown in Table 1. In general, the running time of MJ2 and AVI is longer than that of MP4. The running time of MP4 with time‐prioritized sequence is similar to that of MP4 with location‐prioritized sequence. The compression ratios of MJ2 and AVI are far less than that of MP4. MP4 with location‐prioritized sequence has a higher compression ratio than that of MP4 with time‐prioritized sequence. The means and standard deviations of DIFF of time‐prioritized sequence are larger than those of location‐prioritized sequence. The means and standard deviations of CORR of time‐prioritized sequence are similar to those of location‐prioritized sequence. Among three treatment sites, compression ratio is highest for head‐and‐neck cases, medium for thorax cases, and lowest for pelvis cases.

**Table 1:** Comparison of compression performance for three video compression algorithms (MJ2, AVI, and MP4)

SITES	MJ2		AVI		MP4							
					Time‐Prioritized Sequence				Location‐Prioritized Sequence			
	CR	T (ms)	CR	T (ms)	CR	T (ms)	DIFF	CORR	CR	T (ms)	DIFF	CORR
HN	15.43	28.25	72.60	81.16	516.54	4.94	5.51 ± 25.18	0.86 ± 0.16	549.87	4.87	4.25 ± 16.96	0.85 ± 0.16
THORAX	18.14	24.93	10.51	89.87	229.00	4.29	10.18 ± 56.06	0.75 ± 0.18	304.17	4.33	6.91 ± 36.41	0.74 ± 0.17
PELVIS	9.22	12.32	6.31	88.97	102.29	3.55	5.58 ± 47.20	0.88 ± 0.18	121.68	3.56	5.36 ± 30.13	0.86 ± 0.17

### 3.2 Video decompression

The performance of three video decompression algorithms was compared as shown in Table 2. For MJ2, there is no image loss due to the nature of lossless compression algorithm. The running time is nearly constant with respect to different video decompression algorithms and treatment sites. For two lossy compression algorithms, AVI shows less image loss than that of MP4. The MSE and VQM of AVI are less than those of MP4, while the PSNR of AVI is less than that of MP4. The MSE and VQM of MP4 with time‐prioritized sequence are slightly larger than those of MP4 with location‐prioritized sequence. The PSNR of MP4 with time‐prioritized sequence is similar to that of MP4 with position‐prioritized sequence. Among three treatment sites, MSE is highest for HN cases, medium for thorax cases, and lowest for pelvis cases. PSNR is highest for thorax cases, medium for pelvis cases, and lowest for HN cases. VQM is highest for HN cases, medium for pelvis cases, and lowest for thorax cases.

**Table 2:** Comparison of decompression performance for three video compression algorithms (MJ2, AVI, and MP4)

SITES	MJ2		AVI			MP4							
						Time‐prioritized sequence				Location‐prioritized sequence			
	T (ms)	T (ms)	MSE	PSNR	VQM	T (ms)	MSE	PSNR	VQM	T (ms)	MSE	PSNR	VQM
HN	73.1	69.1	72.60	81.16	14.3E‐5	58.7	968.42	67.87	88.5E‐5	60.9	940.55	68.29	82.2E‐5
THROAX	68.1	70.5	10.51	89.87	3.89E‐5	64.9	368.37	72.81	43.6E‐5	67.2	341.12	73.08	41.5E‐5
PELVIS	75.9	75.6	6.31	88.97	3.34E‐5	66.4	460.60	70.40	54.6E‐5	63.6	432.62	70.65	52.4E‐5

### 3.3 Registration accuracy

The discrepancy of positioning accuracy before and after compression for two lossy algorithms, AVI and MP4, are summarized in Table 3. On average, the mean value of discrepancies between target offsets before and after compression is −0.01 mm ± 0.35 mm. The columns of AVI_T and AVI_P represent the result of AVI encoder using time‐prioritized and position‐prioritized sequences, while MP4_T and MP4_P represent the result of MP4 using these two sequences. The columns of bone or soft tissues represent the result of registration based on bony structures or soft tissues as shown in Figs. 4 and 5. For two lossy compression algorithms, AVI and MP4, their mean values and standard deviations of discrepancies are similar and smaller. It was also noticed that the difference between the results of boney‐structure based and soft‐tissues based registrations are small. The discrepancies of target offsets using time‐prioritized sequences and position‐prioritized sequences are also similar and smaller.

**Table 3:** Comparison of positioning accuracy before and after compression for two lossy video compression algorithms (AVI and MP4)

Dimensions	AVI_T		AVI_P		MP4_T		MP4_P	
	Bone	Soft tissue	Bone	Soft tissue	Bone	Soft tissue	Bone	Soft tissue
Lat (mm)	0.04 ± 0.45	−0.04 ± 0.38	−0.03 ± 0.35	−0.04 ± 0.39	0.08 ± 0.43	−0.08 ± 0.38	0.01 ± 0.45	0.00 ± 0.28
Lng (mm)	−0.05 ± 0.25	−0.04 ± 0.27	−0.03 ± 0.26	−0.03 ± 0.28	−0.03 ± 0.49	−0.05 ± 0.40	−0.04 ± 0.55	−0.02 ± 0.37
Vrt (mm)	0.08 ± 0.55	−0.02 ± 0.16	0.09 ± 0.45	0.02 ± 0.16	0.01 ± 0.48	−0.05 ± 0.16	0.00 ± 0.45	−0.04 ± 0.18

## 4 DISCUSSIONS

MP4 demonstrated its superior compression capability over MJ2 (lossless encoding algorithm) and AVI (lossy intra‐frame coding algorithm). This is attributed to its algorithm in reducing more redundant information between two successive images. The compression ratio of MP4 with position‐prioritized sequence is higher than that of MP4 with time‐prioritized sequence. It was also observed that DIFF of position‐prioritized sequence is less than that of time‐prioritized sequence. Both facts indicate that position‐prioritized sequence may improve the compression ratio for MP4. For different treatment sites, the compression ratios are varied to certain degrees. The compression ratio is highest for head‐and‐neck cases and lowest for pelvis cases for all three video compression algorithms. This implies that the variation of CBCT images between different sessions in head‐and‐neck cases may be less than that in pelvis cases. For those cases with larger variation between different sessions, the compression ratio using video compression algorithm might be lower.

Image loss caused by MP4 compression algorithm is larger than that of AVI in terms of MSE. The MSE of MP4 is nearly 10 times of AVI. But if taking into account the amount of bits of a pixel, this error is smaller. For CBCT image with 16 bits per pixel, if MSE = 900 the relative error per pixel is $\sqrt{\frac{900}{{\left(2^{16}-1\right)}^{2}}}<0.0005$. It was also demonstrated by the fact that PSNR (or VQM) of MP4 is close to that of AVI even if MP4 has larger MSE than AVI. Also note that the MP4 function provided by Matlab is based on H.264 standard which was primarily designed for video transmitting with lower image quality. Its successor, H.265, will provide the substantially improved video quality at the same bit rate.^23^ If H.265 is applied, the image loss due to MP4 algorithm would be further reduced. It was observed that the MSE of head‐and‐neck cases is higher than those of thorax and pelvis cases. This may be caused by the higher compression ratio of head‐and‐neck cases comparing to that of thorax and pelvis cases. For all tested cases, PSNR is in the range of (60–90) and VQM is less than 1% which indicates that the fidelity of decompressed images is well preserved. The registration accuracy based on decompressed CBCT is similar to that of original CBCT without compression. Although there are image losses due to video compression algorithms, its impact on positioning accuracy is hardly discernible with respect to image sequence types, image intensity ranges and compression algorithms. Since both lossy video compression algorithms demonstrated similar positioning accuracy with decompressed CBCT images, it is favorable to use MP4 as it provided higher compression ratio.

Considering the time cost of video compression, AVI takes the most time while MP4 uses the least time. This may be caused by the variation of computation times of three video compression algorithms. For decompression process, the running times of three video compression algorithms are close. Although the running time of video compression algorithm is varied, the absolute value of running time is smaller. For a CBCT set consisting of 1000 images, it takes 10 seconds for compression and 60 seconds for decompression using MP4 algorithm. In addition to the higher compression ratio, there is another advantage in compressing CBCT using video compression algorithm, i.e., the resulting movie can be quickly viewed. The common image compression algorithm placed all images in a folder and generated a zip file for all images in the folder. The image content in the zip file could not be viewed unless it was unzipped. With video compression algorithm, the resulting movie file can be viewed freely without decompression process. This is a huge benefit for many medical image applications.

## 5 CONCLUSION

Video compression method is an effective way for the repository of clinical CBCT data. The lossless video compression method provides lower compression ratio but higher image quality, while the lossy compression method provides higher compression ratio but lower image quality. The selection of image sequences will change compression performance of those video compression algorithms which employ inter‐frame coding algorithm. For patient positioning, MP4 is the most suitable method for CBCT image compression among three video encoders because it has highest compression ratio and comparable positioning accuracy. As video compression methods may cause image loss, it should be cautious to apply them for those clinical applications which required higher quality of image detail.

## CONFLICT OF INTEREST

The authors have no relevant conflicts of interest to disclose.
